# From digitalization to nutrition: the role of digital village construction in shaping dietary intake of rural residents in China

**DOI:** 10.3389/fnut.2025.1709105

**Published:** 2025-12-19

**Authors:** Jiawei Wang, Xia Kuang, Zhihua Wu, Feng Ye, Wenmei Liao

**Affiliations:** 1 School of Health Management, Nanchang Medical College, Nanchang, China; 2 School of Economics and Management, Jiangxi Open University, Nanchang, China; 3 School of Economics, Jinan University, Guangzhou, China; 4 School of Economics and Management, Jiangxi Agricultural University, Nanchang, China

**Keywords:** digital village, nutritional intake, food consumption, digital economy, rural residents

## Abstract

**Introduction:**

As China’s dietary structure shifts from “eating enough” to “eating well and eating healthily,” improving the nutritional status of rural residents through digital development has become a key issue for advancing rural revitalization and common prosperity

**Methods:**

Using data from the 2020 China Rural Revitalization Survey (CRRS), this study empirically examines the impact of digital village construction on rural residents’ nutritional intake and its underlying mechanisms.

**Results:**

The results show that digital village construction significantly improves the nutritional intake of rural residents, with the digital environment playing the most prominent role. Further analysis reveals that digital village construction has the strongest promoting effect on dairy consumption, while its impact is more pronounced in poor villages and non-plain areas, highlighting the importance of digital development in addressing regional disparities and promoting nutritional equity. Mechanism tests indicate that digital village construction improves rural residents’ nutritional intake by enhancing information accessibility, increasing household income, and improving infrastructure.

**Discussion:**

Based on these findings, priority should be given to improving network infrastructure, developing rural e-commerce and cold-chain logistics systems, and strengthening the digital dissemination of nutrition and health knowledge, in order to comprehensively enhance the nutritional status of rural residents.

## 1 Introduction

With the acceleration of urbanization in China, the overall dietary quality of residents has improved, and dietary patterns have gradually shifted from being dominated by grains and vegetables to being centered on high-quality proteins such as meat, eggs, and dairy products (1, 2). This transformation not only reflects rising income levels but also embodies changes in consumption concepts and lifestyles. However, the urban–rural gap remains significant, and malnutrition continues to be a prominent issue in less developed areas. According to the (51)released by the Chinese Nutrition Society, the prevalence of malnutrition, anemia, and vitamin A deficiency among rural residents is higher than that of urban residents (3). Nutritional inadequacy not only affects individual health but also constrains labor quality and rural economic development. Therefore, improving rural nutrition has become an important issue of concern for both the Chinese government and the global community (4). In recent years, the construction of digital villages has provided a new pathway to address this challenge. By enhancing information access, expanding consumption channels, and optimizing resource allocation, digital technologies are reshaping the production and lifestyles of rural residents, thereby creating favorable conditions for dietary optimization and nutritional improvement (5). By the end of 2023, the rural internet population in China had reached 326 million, with an internet penetration rate of 66.5% (6). Services such as internet-based healthcare, rural e-commerce, and digital agriculture have developed rapidly, creating new opportunities to improve dietary quality among rural residents (7, 8). However, due to the lack of micro-level data, it remains unclear how exactly digital village construction affects nutritional intake in transitional economies. For China, as such a transitional economy, digital village construction plays a key role in promoting rural economic transformation and improving residents’ quality of life.

Academic research on nutritional intake has formed a relatively systematic context. Early studies focused on the food environment and accessibility, emphasizing the fundamental role of external supply conditions in shaping dietary structures. For example, improved transportation has reduced food transportation costs and expanded the range of food choices for residents (9); the upgrading of market infrastructure and retail formats has significantly enhanced food diversity and nutritional density by introducing supermarkets and formal channels (10, 11). However, facts have shown that relying solely on improvements in the supply side is difficult to eliminate undernutrition. In some rural areas, even with better market access, low-income households still face constraints on purchasing power and consumption preferences. Combined with the dual effects of price volatility and income limitations, their consumption of high-nutrition and high-protein foods is particularly vulnerable to suppression. When the prices of meat or dairy products rise, such households often respond by reducing consumption or switching to lower-priced substitutes. Consequently, income levels further determine the extent to which dietary structures can shift from “quantity-oriented” to “quality-oriented” consumption (12, 13). On this basis, scholars have turned to the institutional environment and policy intervention. Research has found that the modernization of the retail system has improved food circulation and also increased the intake of protein and micronutrients among children and vulnerable groups (14); food fortification policies have effectively alleviated vitamin deficiency and anemia problems (15, 16); nutritional education and health interventions have enhanced families’ nutritional awareness in food selection (17, 18). These results indicate that improving nutrition not only depends on market conditions but also requires the coordination of institutions and policies. Further research emphasizes the importance of family endowments and individual preferences. Population aging exposes the rural elderly population to the risk of insufficient protein intake (19); family structure and resilience affect their ability to adjust their diet under external shocks (20); and psychological factors such as income expectations, wellbeing, and dietary orientation have also promoted dietary upgrading to varying degrees (21). This type of research expands the explanatory framework for improving nutrition and highlights the role of demand-side factors.

With the advancement of the digital process, the academic community has begun to focus on the relationship between digital rural construction and nutritional intake. Relevant research is mainly divided into two categories: one emphasizes the direct role of the Internet and digital technologies in improving diet quality through the dissemination of nutritional information, convenient transactions, and telemedicine (5, 22, 23); the other emphasizes institutional innovation, arguing that digital infrastructure, digital literacy, and inclusive finance jointly promote consumption upgrading and indirectly improve nutritional status (24–27). Overall, digital rural construction mainly affects nutritional intake through three channels: in terms of the information channel, the Internet and digital platforms have expanded farmers’ access to food prices, nutritional knowledge, and health information, reducing information asymmetry (5, 23); in terms of the income channel, the development of e-commerce and the upward flow of agricultural products have increased farmers’ income, and non-agricultural employment and skill improvement have further enhanced families’ ability to purchase high-nutrition foods (25, 26); in terms of the infrastructure channel, the penetration of e-commerce and express delivery services has enabled residents in remote areas to access fresh and diverse food ingredients more conveniently, thereby improving dietary diversity and nutritional density (22, 27). These three mechanisms jointly supplement and strengthen the role of traditional supply-side environmental improvements, providing a new perspective for explaining nutritional differences in transitional economies.

Nevertheless, there are still several deficiencies in the existing literature. First, most of the literature focuses on consumption structure or income distribution, lacking a systematic analysis of how the construction of digital villages directly affects nutritional intake. Second, there is still a lack of research on the differences in the effects of different dimensions within the construction of digital villages and their heterogeneous impacts under different food categories and village characteristics. Finally, although existing studies have proposed that information acquisition, income improvement, and infrastructure enhancement are the channels through which digitalization works, there is a lack of systematic testing of their relative contributions. In response to the above deficiencies, based on the data from the 2020 China Rural Revitalization Comprehensive Survey (CRRS), this study poses the following research questions: (1) Does digital village construction improve the nutritional intake of rural residents, and to what extent? (2) Do different dimensions of digital village construction vary in their impact on nutrition? (3) Does digital village construction exert heterogeneous effects on different food categories and across villages with different characteristics? (4) Through which mechanisms does digital village construction influence rural residents’ nutritional intake?

The contributions of this study are mainly reflected in three aspects: First, from a research perspective, it integrates digital village construction with nutrition and health, expanding the evaluation framework of digital village outcomes from the angle of nutritional improvement. Second, in terms of content, it not only verifies the overall effect of digital village construction on nutritional intake but also reveals the heterogeneous impacts across construction dimensions, food categories, and village characteristics. Third, in terms of mechanism analysis, it systematically tests the logical effects of the three paths of information acquisition, household income, and infrastructure enhancement, deepening the understanding of the relationship between digital village construction and nutritional improvement. These conclusions not only enrich the relevant academic literature but also provide new policy implications for optimizing the rural diet structure and promoting nutritional balance.

## 2 Theoretical analysis and research hypotheses

With the deepening of the digital economy and the rural revitalization strategy, digital village construction has become an important lever for promoting rural socioeconomic development (28). Its connotation not only includes the improvement of information infrastructure and the diffusion of digital technologies but also extends to the digital transformation of rural governance, public services, and market systems. Against the backdrop of food consumption shifting from “eating enough” to “eating well and eating healthily,” digital village construction plays multiple roles in enhancing the nutritional intake of rural residents. First of all, the improvement of the digital environment has narrowed the digital divide between urban and rural areas, enabling rural residents to access the Internet more conveniently and obtain information on food nutrition and market prices in a timely manner (29). A good network environment not only strengthens residents’ awareness of nutrition but also provides technical support for them to purchase fresh food through online platforms, thus directly promoting the improvement of the nutritional structure and consumption quality. Second, the development of digital services has expanded residents’ channels for obtaining food. With the help of e-commerce platforms, rural residents can more easily access a variety of high-quality foods, including high-nutrition foods such as meat, eggs, and milk, thus breaking through the constraints of local supply in traditional consumption (30, 31). The development of e-commerce has not only increased the availability of nutritious foods but also reduced transaction costs through economies of scale, improving residents’ consumption experience. Finally, the popularization of digital life has deeply integrated farmers’ daily lives into the digital world (32). Smart terminals not only provide convenience for residents to obtain nutrition and health information but also significantly reduce the search and acquisition costs of high-quality foods through emerging consumption models such as group buying, food delivery, and instant delivery. As a result, rural residents consume high-nutrition foods more frequently in their daily lives, and their nutritional levels have improved.

In addition to these direct effects, digital village construction may also promote nutritional intake through several indirect mechanisms. First, the information acquisition mechanism. According to the view of information economics and diffusion of innovation theory (33, 34), information asymmetry and delays in knowledge dissemination often lead to inefficiencies and biases in individual consumption choices. The construction of digital villages has improved the rural information environment through Internet infrastructure and digital platforms, enabling farmers to more conveniently access knowledge about food nutrition, market prices, and healthy diets, and reducing information search costs and cognitive barriers. At the same time, the interactive dissemination of social media and online communities has strengthened the diffusion of the concept of healthy diets and the social learning effect, thus guiding residents to form a more scientific and rational dietary structure. Second, the income improvement mechanism. According to Engel’s law and behavioral economics theory (35, 36), income level and budget constraints directly affect individuals’ food choices and consumption preferences. The construction of digital villages not only improves the income structure by promoting the upward flow of agricultural products and increasing farmers’ operating income, but also expands wage and property income by driving the transfer of rural labor, entrepreneurship, and employment opportunities through the digital industry. The increase in income eases the budget constraint, enabling rural residents to have stronger purchasing power to buy high-quality foods such as meat, eggs, and milk, thus improving their nutritional intake. Third, the infrastructure improvement mechanism. The theory of consumption availability points out (37, 38) that market accessibility and supply-chain efficiency are the core conditions affecting the optimization of the consumption structure. The construction of digital villages has promoted the development of rural infrastructure and cold-chain systems, improved the efficiency of food circulation between urban and rural areas, and shortened the time cost of obtaining fresh ingredients. Especially in areas with poor transportation, it has significantly increased the availability of high-nutrition foods, expanded the food selection space for rural residents, and thus improved the level of nutritional intake.

In summary, digital village construction not only directly enhances the nutritional intake of rural residents but also indirectly exerts influence by improving information access, raising income levels, and optimizing infrastructure conditions. Based on the above analysis, the following hypotheses are proposed:

*Hypothesis 1*: Digital village construction improves the nutritional intake of rural residents.

*Hypothesis 2*: Digital village construction enhances the nutritional intake of rural residents by strengthening information access, increasing income levels, and improving infrastructure conditions.

## 3 Research design

### 3.1 Data source

This study is based on the 2020 wave of the China Rural Revitalization Survey (CRRS), a large-scale national panel survey initiated by the Institute of Rural Development, Chinese Academy of Social Sciences. The survey covers multiple dimensions, including agricultural production, rural development, household livelihoods, and social welfare, and is widely recognized for its representativeness. The sampling design followed a multistage, stratified random procedure. First, the survey team comprehensively considered the economic development level, regional location, and agricultural development situation, and randomly selected 10 provinces from the eastern, central, western, and northeastern regions, respectively, at a ratio of one-third. These provinces are Guangdong Province, Zhejiang Province, Shandong Province, Anhui Province, Henan Province, Guizhou Province, Sichuan Province, Shaanxi Province, Ningxia Hui Autonomous Region, and Heilongjiang Province. Second, the survey team divided all counties (cities and districts) in the sample provinces into five groups equally according to the per-capita GDP level. While considering the geographical spatial distribution, one county (city, district) was randomly selected from each group, that is, five counties (cities and districts) were selected from each sample province. Third, according to the similar sampling principle mentioned above, three townships were randomly selected from each sample county (city and district), and one administrative village with better and one with poorer economic development were randomly selected from each sample township. Finally, according to the equidistant sampling method, 12–14 rural households were randomly selected from the roster provided by the village committee, and on-site surveys were carried out strictly in accordance with the survey plan. In this study, relevant abnormal and missing observations were removed, and finally, 2,133 rural household samples were obtained.

### 3.2 Variable selection

#### 3.2.1 Dependent variable: nutritional intake

This study measures the nutritional intake of rural residents by the share of meat, egg, and dairy purchases in total food consumption. Unlike most existing studies that focus on the intake of macronutrients such as protein, fat, and carbohydrates (39), we emphasize the acquisition of high-quality protein and key micronutrients. Meat, eggs, and dairy products are major sources of high-quality protein and essential micronutrients (e.g., iron, zinc, calcium, and vitamin B₁₂) and have been widely used to evaluate dietary structure and nutritional improvement (40, 41). Previous research indicates that the consumption of animal-sourced foods is highly correlated with nutritional status and health outcomes, particularly in rural areas, where the proportion of animal protein effectively reflects dietary quality improvements and nutritional supply (42). The CRRS collected data on household food consumption using a 30-day recall method, covering grains, soy products, meat, eggs, dairy, and vegetables, which provides a comprehensive view of household dietary patterns. The indicator of nutritional intake used here is defined as the total purchases (in kilograms) of meat (mainly pork, chicken, fish, beef, and lamb), eggs (primarily chicken eggs), and dairy products (including liquid milk and milk powder) divided by total household food purchases (in kilograms). This ratio primarily reflects rural residents’ intake of high-quality protein derived from animal-sourced foods, which serves as a key indicator of improvements in dietary quality and nutritional adequacy in the context of rural nutrition studies.

#### 3.2.2 Independent variable: digital village construction

There is no universally established framework for evaluating digital village construction, as definitions and indicators vary across studies. Drawing on existing research (43) and considering China’s practical context and data availability, we measure digital village construction using three dimensions: digital environment, digital services, and digital life. The digital environment is measured by the village’s broadband penetration rate and overall network condition. The latter is aggregated at the village level based on household reports on internet quality (1 = stable, 0 = occasional or frequent disconnections). Digital services are captured by the number of e-commerce households in the village and the average online sales value, calculated at the village level. Digital life is proxied by the average number of smartphones per household and average daily smartphone usage time, both aggregated to the village level. To ensure the objectivity and scientific validity of the indicator system, this study employs the entropy weighting method to assign weights to the secondary indicators. This method uses the information entropy of each indicator to reflect its degree of variability—indicators with greater variability contribute more to the overall evaluation. In this way, the method objectively determines the weights and avoids biases associated with subjective assignment. The specific variables and weight assignments are presented in Table 1.

**Table 1:** Indicators of digital village construction.

Primary dimension	Secondary indicator	Unit	Direction	Weight	Mean	SD
Digital environment	Village broadband penetration rate	%	+	0.029	0.604	0.346
	Village network condition	−	+	0.018	5.468	2.511
Digital services	Number of e-commerce households in the village	Households	+	0.304	3.87	15.779
	Average online sales per village	10,000 yuan	+	0.616	515.313	4098.391
Digital life	Average number of smartphones per village	Units	+	0.011	32.359	9.383
	Average smartphone usage time per village	Hours	+	0.022	27.095	12.804

#### 3.2.3 Mediating variables

Three mediators are considered: Information accessibility, Household income, and Infrastructure. Information accessibility reflects the ease with which households obtain daily information via mobile phones or the internet, capturing the role of digitalization in information dissemination and knowledge acquisition. Household income measures family income levels, reflecting the potential economic effect of digital village construction on nutritional intake. Infrastructure indicates whether express delivery services are available in the village, reflecting how improvements in digital infrastructure affect residents’ access to diversified food channels. Together, these variables capture three dimensions—information, economic capacity, and circulation channels—through which digital village construction may influence nutritional intake.

#### 3.2.4 Instrumental variable

We use the Digital (52), jointly released by the New Rural Development Research Institute of Peking University and the Ali Research Institute, as the instrumental variable. On the one hand, this index measures county-level digitalization across dimensions such as e-commerce penetration, logistics delivery, and internet infrastructure, making it closely related to village-level digital village construction (relevance criterion). On the other hand, as a county-level macro indicator largely determined by regional digital industries and policy environments, it is unlikely to directly affect household-level nutritional intake (exogeneity criterion).

#### 3.2.5 Control variables

Following prior literature, we incorporate control variables at three levels: household head, household, and village. Household head characteristics include age, gender, education level, and marital status. Household characteristics include household size, dependency ratio, farm size, and self-produced safe food (whether the household produces vegetables or pork for self-consumption). Village characteristics include road condition, distance to the county seat, industrial pollution, and GDP per capita (Table 2).

**Table 2:** Descriptive statistics of variables.

Variable name	Definition/coding	Mean	SD
Nutritional intake	Share of meat, egg, and dairy consumption (kg) in total food consumption (kg).	0.178	0.131
Digital village construction	Composite indicator constructed from digital village development measures.	1.676	0.599
Information accessibility	Access to daily information via mobile phone or internet (1 = difficult; 2 = sometimes; 3 = fully accessible).	2.235	0.839
Household income	Annual household income in 2019, log-transformed.	1.798	0.836
Infrastructure	Availability of express delivery services in the village (1 = yes; 0 = no).	10.794	1.065
Digital village index	County-level Digital Village Index of China in 2020 (official data source).	59.021	13.981
Age	Household head’s Age (years).	54.791	10.568
Gender	Household head’s gender (1 = male; 0 = female).	0.945	0.228
Education level	Household head’s education level (1 = no schooling; 2 = primary school; 3 = junior high school; 4 = senior high school; 5 = technical secondary school; 6 = vocational/technical school; 7 = college diploma; 8 = bachelor’s degree; 9 = postgraduate degree).	2.819	1.029
Marital status	Household head’s marital status (1 = married; 0 = otherwise).	0.935	0.246
Household size	Number of individuals who had meals at home during the past month.	3.348	1.609
Dependency ratio	Ratio of non-working-age individuals (aged 0–14 and 60+) to working-age individuals (aged 15–59) among those who had meals at home during the past month.	0.368	0.355
Farm size	Total cultivated land area of the household (mu).	21.693	70.612
Self-produced safe food	Whether the household produces some “safe” vegetables/pork for its own consumption (1 = yes; 0 = no).	0.709	0.454
Road condition	Whether the road between the village and the group is paved (1 = yes; 0 = no).	0.946	0.227
Distance to county seat	Distance from village committee to county government (km).	5.672	5.608
Industrial pollution	Whether the village experienced industrial pollution in the past five years (1 = yes; 0 = no).	0.075	0.264
GDP per capita	Natural logarithm of county-level per capita GDP (yuan per person).	1.755	0.611

### 3.3 Model construction

The dependent variable in this study is the nutritional intake level of rural residents, measured by the animal-based food ratio (the share of meat, egg, and dairy consumption in total food consumption). Since this ratio ranges between [0,1], applying OLS directly may yield predicted values outside the feasible range. To address this, we adopt the Fractional Logit Model proposed by Papke and Wooldridge. The conditional expectation function is specified as:


$$E(Y_{i}|X_{i})=G(\alpha +\text{βDigtalVillag}e_{i}+\mu X_{i})$$ (1)

where $Y_{i}$ denotes the nutritional intake level of rural residents, $\text{DigtalVillag}e_{i}$ represents the level of digital village construction, $X_{i}$ is the vector of control variables, and α,β,μ are parameters to be estimated. The logistic function is defined as:


$$G(z)=\frac{e^{z}}{1+e^{z}}$$ (2)

This specification ensures that predicted values always lie within the interval (0,1). Parameter estimation is conducted within the Generalized Linear Model (GLM) framework using Bernoulli Quasi-Maximum Likelihood Estimation (QMLE), with robust standard errors applied to control for heteroscedasticity.

To further examine the mechanisms through which digital village construction affects rural residents’ nutritional intake, we follow existing research (44) and construct a mediation model as follows:


$$M_{i}=\gamma +\text{θDigtalVillag}e_{i}+\delta X_{i}+\varepsilon_{i}$$ (3)

where $M_{i}$ denotes the mediating variables, including information accessibility, infrastructure, and household income; γ,θ,δ are parameters to be estimated; and $\varepsilon_{i}$ is the error term. Other variables are defined as in Equation 1.

## 4 Results and analysis

### 4.1 Benchmark regression results

The regression results of digital village construction on rural residents’ nutritional intake are presented in Table 3, with the model specifications corresponding to Equations 1, 2. Column (1) reports the univariate regression of digital village construction on nutritional intake, while columns (2), (3), and (4) gradually incorporate control variables at the household head, household, and village levels, respectively. The results show that, regardless of whether control variables are included, the impact of digital village construction on nutritional intake remains significantly positive at the 1% level. This confirms Hypothesis 1, namely, that digital village construction significantly enhances rural residents’ nutritional intake.

**Table 3:** Benchmark regression results.

Variable	(1)	(2)	(3)	(4)
	Nutritional intake	Nutritional intake	Nutritional intake	Nutritional intake
Digital village construction	0.433^***^ (0.031)	0.406^***^ (0.031)	0.399^***^ (0.032)	0.393^***^ (0.032)
Age		−0.008^***^ (0.002)	−0.009^***^ (0.002)	−0.009^***^ (0.002)
Gender		−0.197^**^ (0.088)	−0.194^**^ (0.087)	−0.180^**^ (0.088)
Education level		0.087^***^ (0.019)	0.081^***^ (0.019)	0.076^***^ (0.019)
Marital status		0.073 (0.082)	0.082 (0.082)	0.078 (0.081)
Household size			0.017 (0.011)	0.013 (0.011)
Dependency ratio			0.082 (0.059)	0.073 (0.059)
Farm size			−0.000 (0.000)	−0.000 (0.000)
Self-produced safe food			−0.189^***^ (0.042)	−0.177^***^ (0.042)
Road condition				−0.020 (0.089)
Distance to county seat				−0.008^**^ (0.003)
Industrial pollution				0.175^***^ (0.059)
GDP per capita				−0.015 (0.031)
Constant	−2.278^***^ (0.058)	−1.935^***^ (0.165)	−1.804^***^ (0.169)	−1.702^***^ (0.187)
*N*	2,095	2,095	2,095	2,095

Robust standard errors are reported in parentheses. *, **, and *** denote significance at the 10, 5, and 1% levels, respectively. The same applies to the following tables.

Based on the regression results in column (4), this study further reveals the impact of each control variable on the nutritional intake of rural residents. Specifically, the age of the household head is significantly negatively correlated with nutritional intake, indicating that the increase in age is often accompanied by a more traditional and thrifty diet structure, with insufficient demand for high-quality nutritional products such as meat, eggs, and milk, thus inhibiting the improvement of the nutritional level. On the contrary, the education level has a significant positive effect. Farmers with a higher level of education usually have stronger health awareness and nutritional knowledge, and can actively optimize the diet structure and increase the consumption of high-protein and high-nutrition foods. In terms of the connection between production and consumption, the “safe” self-supplied food of the household has a negative impact on nutritional intake. This may be because self-supplied production is limited to a small amount and a single type of food, and at the same time, it reduces the rural residents’ purchase of high-quality market-supplied foods such as meat, eggs, and milk, which is not conducive to the improvement of the nutritional level. The negative effect of the distance from the county government further shows that the location condition plays an important role in obtaining high-quality foods. Inconvenient transportation directly restricts the rural residents’ accessibility to the diverse market supply. Finally, environmental pollution also has a negative impact on nutritional intake, reflecting its damage to the quality and safety of local agricultural products and the squeezing of rural residents’ health expenditure and consumption ability, ultimately limiting the intake of high-quality foods.

### 4.2 Robustness tests

#### 4.2.1 Alternative model

To verify the robustness of the baseline results, we re-estimated the model using OLS regression. The results, reported in column (1) of Table 4, show that the estimated coefficient of digital village construction remains significantly positive, consistent with the baseline findings.

**Table 4:** Robustness test results.

Variable	(1)	(2)	(3)	(4)
	Nutritional intake	Nutritional intake_1	Nutritional intake	Nutritional intake
Digital village construction	0.061^***^ (0.005)	0.272^***^ (0.029)	0.224^***^ (0.025)	0.439^***^ (0.036)
Control variables	Yes	Yes	Yes	Yes
Constant	0.145^***^ (0.028)	−1.328^***^ (0.162)	−1.272^***^ (0.188)	−1.708^***^ (0.188)
*N*	2,095	2,095	2,095	2,095

#### 4.2.2 Alternative dependent variable

To verify the above-mentioned conclusion, this study replaces the dependent variable with the proportion of rural households’ meat, egg, and milk consumption expenditure in food consumption expenditure (*Nutritional intake_1*) for regression. The results are shown in column (2) of Table 4. The results show that digital rural construction significantly promotes rural residents’ nutritional intake at the 1% level, and the previous conclusion still holds.

#### 4.2.3 Alternative measurement of digital village construction

There are various measurement methods for digital rural construction. This study uses the factor analysis method to conduct a re-measurement. First, two factors with eigenvalues greater than 1 are retained, and the KMO test value is 0.592, indicating good correlation among the measurement items; second, the chi-square value of the Bartlett spherical test is significant at the 1% statistical level, indicating that the factor analysis results are valid. As shown in column (3) of Table 4, digital rural construction has a significant positive impact on rural residents’ nutritional intake, which is consistent with the previous conclusion.

#### 4.2.4 Alternative measurement of digital village construction

To eliminate the potential influence of outliers on the regression results, this study applies the Winsorization method (45) to trim 5% of the extreme values for continuous variables such as age and household size. The regression is then re-estimated, and the results are reported in column (4) of Table 4. After removing the impact of extreme observations, the coefficient of digital village construction remains significantly positive at the 1% level, indicating that the core conclusions are robust.

### 4.3 Endogeneity test

Although the robustness checks above strengthen the credibility of the baseline results, potential endogeneity concerns cannot be fully ruled out. First, some unobserved or uncontrolled factors may simultaneously affect both digital village construction and nutritional intake, leading to biased estimates. Second, since digital village construction is measured as a composite indicator, potential measurement errors in its construction process may compromise the accuracy of the explanatory variable. Third, digital village construction projects may be prioritized in economically developed, more populous, or policy-favored regions, where residents’ nutritional conditions are already better than elsewhere, which could introduce sample selection bias.

To address these issues, we use the 2020 County-level Digital Village Index as an instrumental variable. The regression results are reported in Table 5. As shown in column (1), the Digital Village Index has a significant positive effect on digital village construction, with a first-stage *F*-statistic of 45.50 indicating that weak instrument problems are unlikely. Moreover, the Wald exogeneity test rejects the null hypothesis of “no endogeneity in digital village construction” at the 1% significance level. Column (2) shows that, after accounting for endogeneity, digital village construction still exerts a significant positive effect on the nutritional intake of rural residents.

**Table 5:** Endogeneity test results.

Variable	(1)	(2)
	Digital village construction	Nutritional intake
Digital village index	0.017^***^ (0.001)	
Digital village construction		0.143^***^ (0.013)
Control variables	Yes	Yes
Constant	0.129 (0.118)	0.091^***^ (0.030)
First stage *F*-value	45.50	
*N*	2,095	2,095

### 4.4 Further analysis

Based on the previous baseline regression and mechanism analysis, this study further conducts an analysis from three perspectives: different dimensions of digital rural construction, types of food for nutritional intake, and differences in village characteristics, so as to more comprehensively reveal the impact of digital rural construction on the nutritional intake of rural residents.

#### 4.4.1 Dimensions of digital rural construction

In the previous variable construction, this study divides digital rural construction into three dimensions: digital environment, digital services, and digital life. To explore the differences in the impact of different dimensions of digital rural construction on the nutritional intake of rural residents, this study conducts regression analysis on each dimension, respectively. The results are shown in Table 6. It can be seen that the digital environment has the most significant positive impact on the nutritional intake of rural residents, followed by digital services, while the role of digital life is relatively small. This indicates that the improvement of network infrastructure is a key prerequisite for improving the nutritional level of farmers. It not only helps farmers obtain information related to nutrition and health, but also creates conditions for the acquisition of diverse foods. At the same time, the development of e-commerce has also broadened the food sources to a certain extent and promoted the consumption of high-nutritional-value foods such as meat, eggs, and milk. In contrast, although the popularization of smart terminals has increased the opportunities for information access and consumption, their role in improving the nutritional structure in the short term is still relatively limited. Therefore, in the process of promoting digital rural construction, priority should be given to improving network infrastructure, and actively promoting the digital application in e-commerce and consumption links, so as to more effectively improve the nutritional intake of rural residents.

**Table 6:** Different dimensions of digital rural construction.

Variable	(1)	(2)	(3)
	Nutritional intake	Nutritional intake	Nutritional intake
Digital environment	1.716^***^ (0.161)		
Digital services		1.601^***^ (0.140)	
Digital life			0.686^***^ (0.057)
Control variables	Yes	Yes	Yes
Constant	−1.558^***^ (0.187)	−1.576^***^ (0.187)	−1.768^***^ (0.187)
*N*	2,095	2,095	2,095

#### 4.4.2 Types of nutritional intake

From the perspective of the heterogeneity of nutritional intake types, this study constructs indicators of the proportion of meat, egg, and milk purchases in the total food consumption, respectively, to examine the impact of digital rural construction on different types of nutritional intake. The regression results in Table 7 show that digital rural construction has a significant positive impact on the proportions of meat, egg, and milk purchases. Among them, the promotion effect on milk is the most significant, followed by meat, and the effect on eggs is the smallest. This may be because the original consumption bases of milk and meat in rural areas were relatively low. Constrained by conditions such as price, supply, and cold-chain logistics, there is more room for improvement. Digital rural construction has improved the circulation channels and information access, significantly reducing the access threshold for milk and meat. Coupled with the improvement of rural residents’ nutritional awareness, the growth in milk and meat consumption is particularly obvious. In contrast, eggs already account for a relatively large proportion of the diet of rural residents. The incremental effect brought by digital construction is relatively limited, so its promotional effect is relatively weak.

**Table 7:** Different types of food consumption.

Variable	(1)	(2)	(3)
	Meat ratio	Egg ratio	Dairy ratio
Digital village construction	0.374^***^ (0.034)	0.182^***^ (0.041)	0.423^***^ (0.055)
Control variables	Yes	Yes	Yes
Constant	−2.149^***^ (0.214)	−3.266^***^ (0.253)	−4.207^***^ (0.414)
*N*	2,095	2,095	2,095

#### 4.4.3 Differences in village characteristics

Considering the differences at the village level, this study conducts a heterogeneity analysis from two dimensions: economic status and topographical conditions. As can be seen from columns (1) and (2) of Table 8, in terms of economic status, the promotion effect of digital rural construction on nutritional intake in poor villages is significantly greater than that in non-poor villages. This indicates that digital development has a stronger marginal effect in areas with relatively scarce resources and can effectively alleviate the constraints of insufficient information and infrastructure conditions on nutritional intake. As can be seen from columns (3) and (4) of Table 8, in terms of topographical conditions, the role of digital rural construction is more prominent in non-plain areas. This shows that in villages with relatively unfavorable transportation and geographical conditions, digital rural construction can more significantly promote residents’ food access and nutritional improvement by improving information circulation and food delivery systems. Overall, the impact of digital rural construction on the nutritional intake of rural residents is more significant in poor villages and non-plain areas, which reflects its important role in making up for regional development shortcomings and promoting nutritional balance.

**Table 8:** Heterogeneity of village characteristics.

Variable	Village economic status		Village topographical features	
	Poor	Non-poor	Plains	Non-plains
	(1)	(2)	(3)	(4)
	Nutritional intake	Nutritional intake	Nutritional intake	Nutritional intake
Digital village construction	0.407^***^ (0.068)	0.364^***^ (0.037)	0.378^***^ (0.043)	0.412^***^ (0.047)
Control variables	Yes	Yes	Yes	Yes
Constant	−1.782^***^ (0.328)	−1.621^***^ (0.235)	−2.820^***^ (0.348)	−1.407^***^ (0.224)
*N*	633	1,462	925	1,170

### 4.5 Mechanism analysis

To explore the paths and mechanisms through which the construction of digital villages affects the nutritional intake of rural residents, this study conducts mechanism tests from three aspects: information availability, household income level, and infrastructure conditions.

#### 4.5.1 Information availability

Information accessibility is not only related to farmers’ attention to food nutrition but also has an important impact on the formation of farmers’ concepts of food nutritional value. Therefore, this study takes information availability as the explained variable and conducts a regression analysis through Equation 3. The results are reported in column (1) of Table 9. The results show that the coefficient of the construction of digital villages is significantly positive, indicating that the construction of digital villages has significantly improved farmers’ information acquisition ability. With the popularization of digital infrastructure and applications, farmers can more quickly grasp the dynamics of market supply and demand and food prices and conveniently obtain knowledge related to nutrition and health. This expansion of information not only improves farmers’ cognition but also gradually changes their consumption concepts and eating habits, making them more inclined to increase the proportion of high-nutrition foods such as meat, eggs, and milk in their food choices, thus optimizing the dietary structure.

**Table 9:** Results of mechanism analysis.

Variable	(1)	(2)	(3)
	Information accessibility	Household income	Infrastructure
Digital village construction	0.245^***^ (0.027)	0.351^***^ (0.041)	0.286^***^ (0.030)
Control variables	Yes	Yes	Yes
Constant	2.117^***^ (0.173)	9.752^***^ (0.215)	0.780^***^ (0.167)
*N*	2,095	2,095	2,095

#### 4.5.2 Household income level

Income status is an important factor affecting the health status of rural residents and also an important determinant of rural residents’ nutritional intake and dietary quality. Therefore, this study takes household income as the explained variable and conducts a regression analysis through Equation 3. The results are reported in column (2) of Table 9. The results show that the construction of digital villages has a significant positive effect on household income. With the in-depth construction of digital villages, the rural production mode is gradually transforming, and employment opportunities are becoming increasingly diverse. Farmers can increase their income through various channels. The increase in income means that the household budget constraint is alleviated, and more resources can be used to purchase high-quality food, thus improving food consumption both in terms of frequency and structure. This income effect not only enhances farmers’ ability to pursue a healthy diet but also objectively promotes the improvement of the nutritional level.

#### 4.5.3 Infrastructure conditions

Rural infrastructure—including transportation, delivery, and cold-chain systems—as a key channel for the flow of urban–rural elements, also affects the quality of food consumption and the nutritional structure of rural residents. Therefore, this study takes infrastructure as the explained variable and conducts a regression analysis through Equation 3. The results are reported in column (3) of Table 9. The results show that the construction of digital villages has significantly improved rural infrastructure conditions. Empowered by digitalization, the delivery system has become more efficient, the time for urban–rural food circulation has been shortened, and the channels for farmers to obtain diverse and high-quality food have been expanded. Especially in remote areas with poor transportation, the improvement of infrastructure conditions is particularly crucial, as it effectively reduces the difficulty of obtaining fresh food, enabling farmers to more conveniently enjoy the supply of the food market. With the improvement of infrastructure conditions, the scope of farmers’ food choices has been significantly expanded, and the nutritional level has been further improved.

The abovementioned analysis shows that the construction of digital villages has a positive impact on the nutritional intake of rural residents by enhancing information acquisition, increasing income levels, and improving infrastructure conditions, verifying Hypothesis 2.

## 5 Discussion and conclusion

### 5.1 Discussion

Based on the previous conclusions, this study further discusses the empirical results in combination with relevant studies. The research results show that digital village construction significantly improves the nutritional intake of rural residents, as measured by the share of meat, egg, and dairy consumption. This result indicates that digital development has played an important role in improving the nutritional intake level of rural residents by improving information channels, expanding consumption methods, and optimizing the diet structure (29, 46). Among different dimensions, the promoting effect of the digital environment is the most prominent, followed by digital services, while the effect of digital life is relatively limited. This reflects that network infrastructure is a prerequisite for promoting nutritional improvement, and the development of e-commerce has broadened the food sources to a certain extent. In contrast, although the popularization of smart terminals has increased information access, it is difficult to directly improve the diet structure in the short term, which is consistent with the relevant views of the “digital divide” theory (47). In terms of food categories, the construction of digital villages has the most significant pulling effect on milk consumption, followed by meat, while the impact on egg consumption is relatively weak. The reason may be that the consumption base of milk and some types of meat in rural areas is relatively low, and they are highly dependent on the cold chain and circulation. Therefore, there is more room for improvement after the improvement of the digital infrastructure (48). In addition, the construction of digital villages has a more obvious effect in poor villages and non-plain areas. This shows that digital development has a stronger marginal effect in areas with scarce resources and inconvenient transportation (49), which helps to make up for development shortcomings and promote nutritional balance. Mechanism analysis shows that the construction of digital villages promotes nutritional improvement mainly through three paths: enhancing information acquisition, increasing household income, and improving infrastructure conditions. This echoes the explanation of information asymmetry in information economics (34), Engel’s law (35), and the theory of diet transformation (50).

Overall, this study not only verifies the positive effect of the construction of digital villages on improving the rural diet structure but also provides new evidence for understanding how digitalization affects nutritional intake through multiple paths. Of course, this study still has certain limitations. First, the CRRS data is based on a 30-day recall, which may underestimate the impact of some long-term consumption patterns. Second, the nutritional intake indicator—measured by the share of meat, egg, and dairy consumption—captures protein intake but does not fully encompass other key nutritional dimensions such as fruits and vegetables. Finally, the study relies on cross-sectional data from the 2020 China Rural Revitalization Survey (CRRS) conducted by the Chinese Academy of Social Sciences, which limits our ability to explore the dynamic evolution and long-term effects of digital village construction. Future research could incorporate data from subsequent years or construct county-household panel datasets to examine, from a dynamic perspective, the sustained impacts, lag effects, and transmission mechanisms of digital village construction on rural residents’ nutritional structures, thereby providing a more comprehensive understanding of how digitalization contributes to long-term improvements in rural wellbeing.

### 5.2 Conclusion

Based on the data of the China Rural Revitalization Comprehensive Survey (CRRS) in 2020, this study systematically analyzes the influencing mechanism of the construction of digital villages on the nutritional intake of rural residents and further examines the impacts of the dimensions of digital village construction, types of nutritional intake, and differences in village characteristics. The main conclusions are as follows: (1) Digital village construction significantly enhances nutritional intake (as measured by the animal-based food ratio), and this finding remains robust after multiple robustness checks and addressing endogeneity. This suggests that digital development not only provides rural residents with more convenient consumption channels and information access but also objectively improves food availability, thereby shifting dietary patterns from “quantity-driven” to “quality-driven.” (2) Among the various dimensions of digital village construction, the promoting effect of the digital environment is the most prominent, followed by digital services, while the impact of digital life is relatively limited. This indicates that network infrastructure is a key prerequisite for promoting rural nutritional improvement, and the development of e-commerce has expanded food sources to a certain extent, but the marginal effect of the popularization of smart terminals is still relatively limited in the short term. (3) The construction of digital villages has different impacts on different types of nutritional foods. It has the most significant promoting effect on milk consumption, followed by meat, while the effect on eggs is relatively weak. This shows that in categories with a low consumption base and strong constraints, such as price and cold-chain, digital development has released greater growth potential. (4) The construction of digital villages has a more prominent effect in poor villages and non-plain areas. This indicates that in areas with scarce resources and inconvenient transportation, digital development can effectively make up for development shortcomings, significantly improve the food availability of residents, and play a more important role in promoting nutritional balance. (5) Digital villages construction significantly promotes the nutritional intake of rural residents mainly through three paths: enhancing information acquisition, increasing household income levels, and improving infrastructure conditions. This not only means that the construction of digital villages has improved consumption conditions but also has fundamentally changed the way of obtaining nutrition, providing long-term impetus for promoting the optimization of rural residents’ diet structure and nutritional improvement.

## Data Availability

The original contributions presented in the study are included in the article/supplementary material, further inquiries can be directed to the corresponding authors.
